# Improvement and effectiveness analysis of dynamic/static imbibition experiments

**DOI:** 10.1371/journal.pone.0310257

**Published:** 2024-10-21

**Authors:** Leilei Zhang, Huiming An, Qing Guo, Yanli Zhang, Li Zhao, Dongliang He, Wenqiang Yang, Cheng Wang

**Affiliations:** Baili College of Petroleum Engineering, Lanzhou City University, Lanzhou, China; China University of Petroleum Beijing, CHINA

## Abstract

In low-permeability fractured reservoirs, there is a generalized fluid displacement between the replacement fluid in the fracture and the matrix crude oil. This imbibition behavior plays a crucial role in the development of this type of reservoir. The experimental devices for studying static imbibition behavior are generally susceptible to air pollution on the surface of the test core and a long testing period; the experimental devices for studying dynamic imbibition behavior are generally unable to eliminate the driving action. A dual-purpose experimental setup and an experimental method for dynamic or static imbibition that can avoid the above defects were designed. A method of fracture fluid flow rate calculation and motor speed conversion is proposed, and the method is used to assist in setting the parameters of dynamic imbibition experiments. The device was applied to compare the experimental effects with the static imbibition bottle and the dynamic replacement imbibition, respectively, and the effect of fracture width on the dynamic imbibition of repellent oil was investigated. The results show that: the static imbibition recovery rate of the dynamic/static imbibition experimental device is 1.55 percentage points higher than that of the imbibition bottle; the dynamic imbibition recovery rate is 3–6 percentage points lower than that of the driving dynamic imbibition method, and it can reflect the influence of a single flow rate condition on the imbibition; imbibition in low-permeability fractured reservoirs is more likely to take place in the fracture in the interval of 50–100 μm in the width.

## 1. Introduction

Spontaneous imbibition induced by capillary pressure in the formation is a widespread and important seepage phenomenon [1–3]. The phenomenon of imbibition in the oil formation is mainly reflected in the process of spontaneous imbibition of oil and water phase fluids in the capillary pore channels [4,5]. The prevalence of oil and water phases in cracks and pore throats of low-permeability fractured reservoirs makes imbibition phenomenon play a crucial role in the development of extra-low-permeability fractured reservoirs [6–8].

The imbibition process in extra-low-permeability fractured reservoirs can be categorized into two types: static imbibition (shut-in imbibition) and dynamic imbibition (drive-in imbibition) [9–12]. At present, most of the literature provides a detailed description of static imbibition, and the information on the quantity distribution of oil and water in large and small pores and throats and the recovery rate of static imbibition in static imbibition rock samples is obtained by the imbibition bottle experimental method in combination with the NMR of the core specimen and the CT scanning of the core slices [13–16]. However, the imbibition bottle experimental method often puts the oil-saturated core directly into the imbibition medium from the air medium, and the air pollution on the core surface (adsorption of air on the core surface) will change the imbibition characteristics of the core surface, which in turn will affect the oil-water displacement and the determination of the volume of surfaced oil [17–23]. Dynamic imbibition research methods are mostly limited to the static imbibition based on additional pulse pressure conditions or experiments using core holder and replacement equipment, and the measured recovery rate covers the dual role of replacement and imbibition, and does not reflect the single imbibition phenomenon under flow conditions [24–27]. In addition, when the same rock sample is subjected to dynamic and static imbibition measurements successively, it is necessary to switch between different experimental devices, and low experimental efficiency and long cycle time are the obvious characteristics of this type of experiments [28–31].

Based on the problems exposed by conventional imbibition experiments, a new type of dynamic and static dual-use imbibition experimental device and experimental method are designed. The device was applied to carry out dynamic and static imbibition experiments on extra-low permeability rocks, respectively, to compare the static imbibition experiments with imbibition bottles, and to compare the dynamic imbibition experiments with driven imbibition, to investigate the differences in the recovery rates of dynamic and static imbibition under the conditions of different experimental devices. In addition, the device was applied to simulate the fluid flow rate in cracks of different widths to investigate the effect of the crack width on the imbibition effect.

## 2. Dynamic/Static imbibition experimental setup and experimental method design

The structure of the device is designed to avoid air contamination of the core surface saturated with oil during the operation of the imbibition experiment. By installing perturbation motors and designing multiple experimental bins in parallel to eliminate the displacement effect generated by the flow rate, we can realize the simultaneous execution of multiple imbibition experiments at different flow rates, thus shortening the experimental cycle and improving the efficiency of the experiments.

### 2.1 Experimental setup design

**Fig 1:**
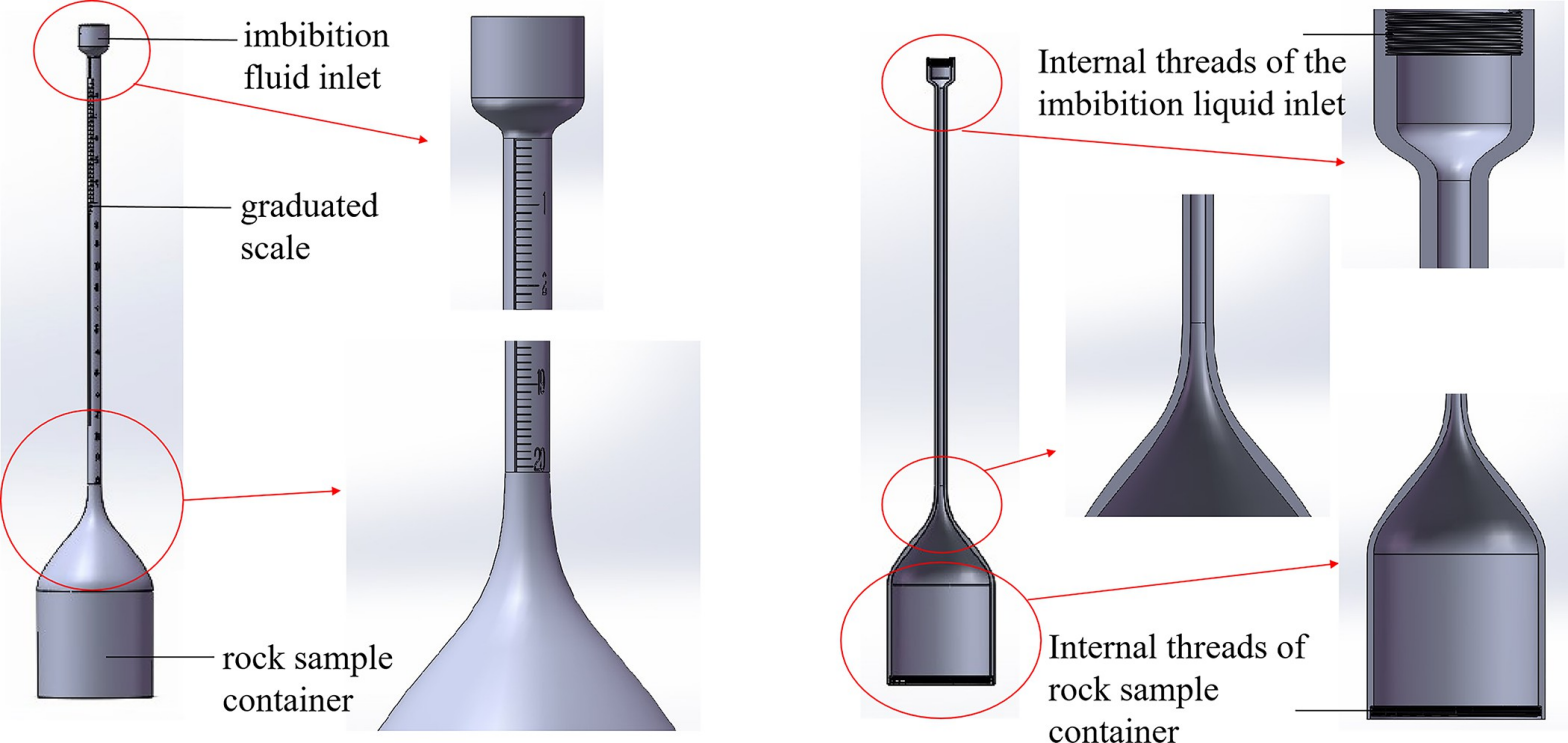
Fine tube measuring tool (inverted graduated tube) and its profile.

**Fig 2:**
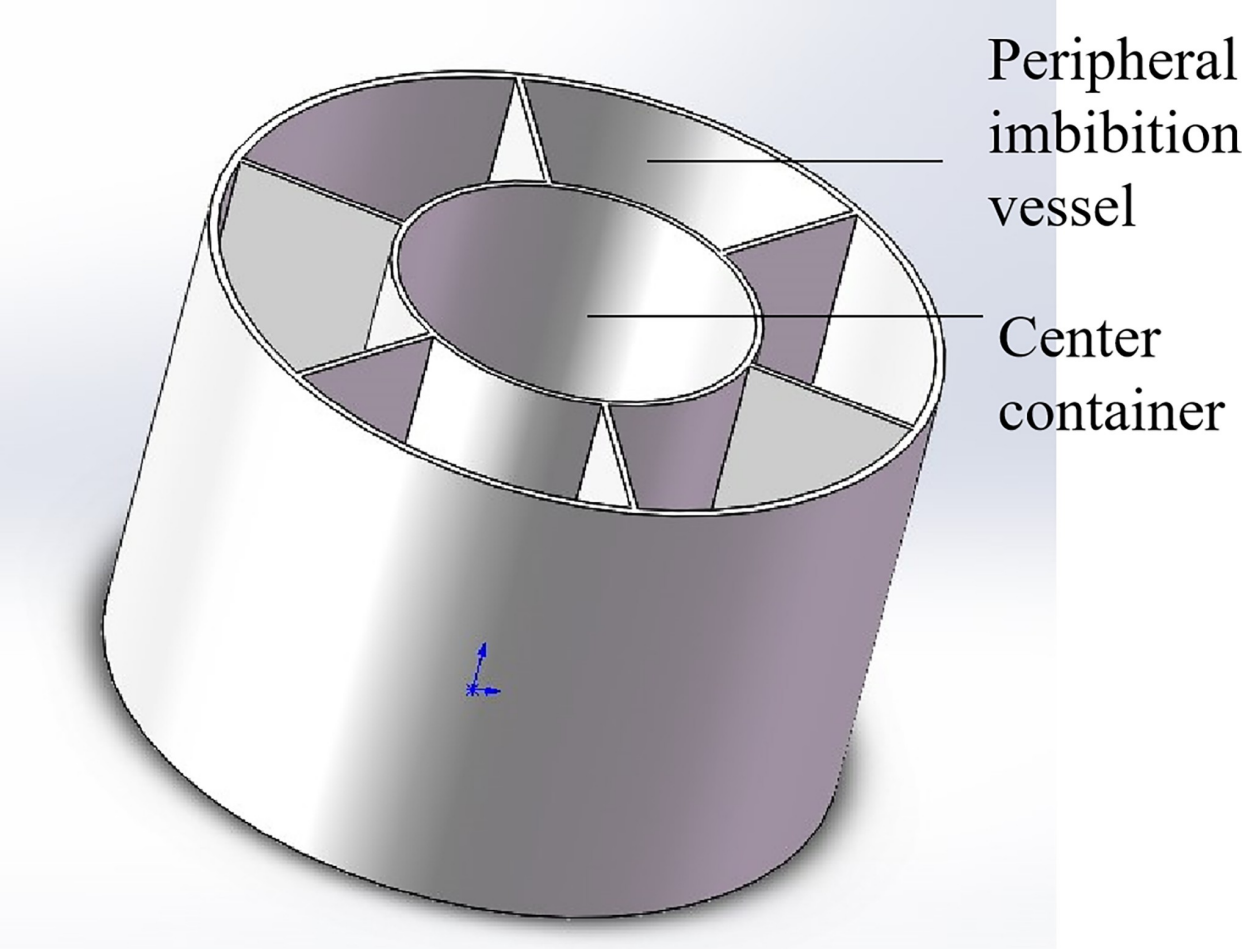
Oil and water container: Six imbibition containers are distributed in a circle; the middle is the central container; and the imbibition container is connected to the bottom of the centralcontainer.

**Fig 3:**
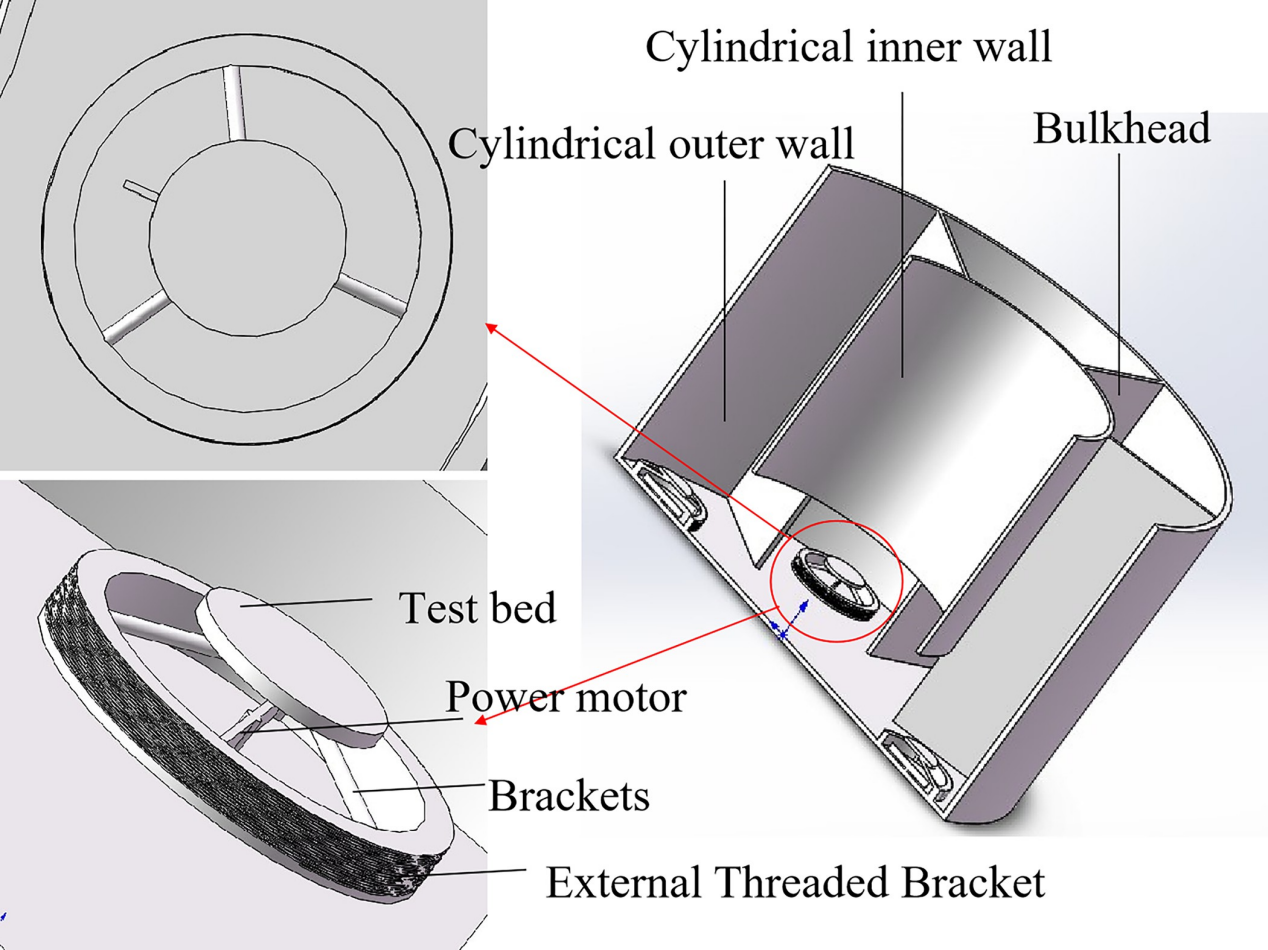
Oil and water container profile and rock sample support platform: The motor is placed above the rock sample support table.

**Fig 4:**
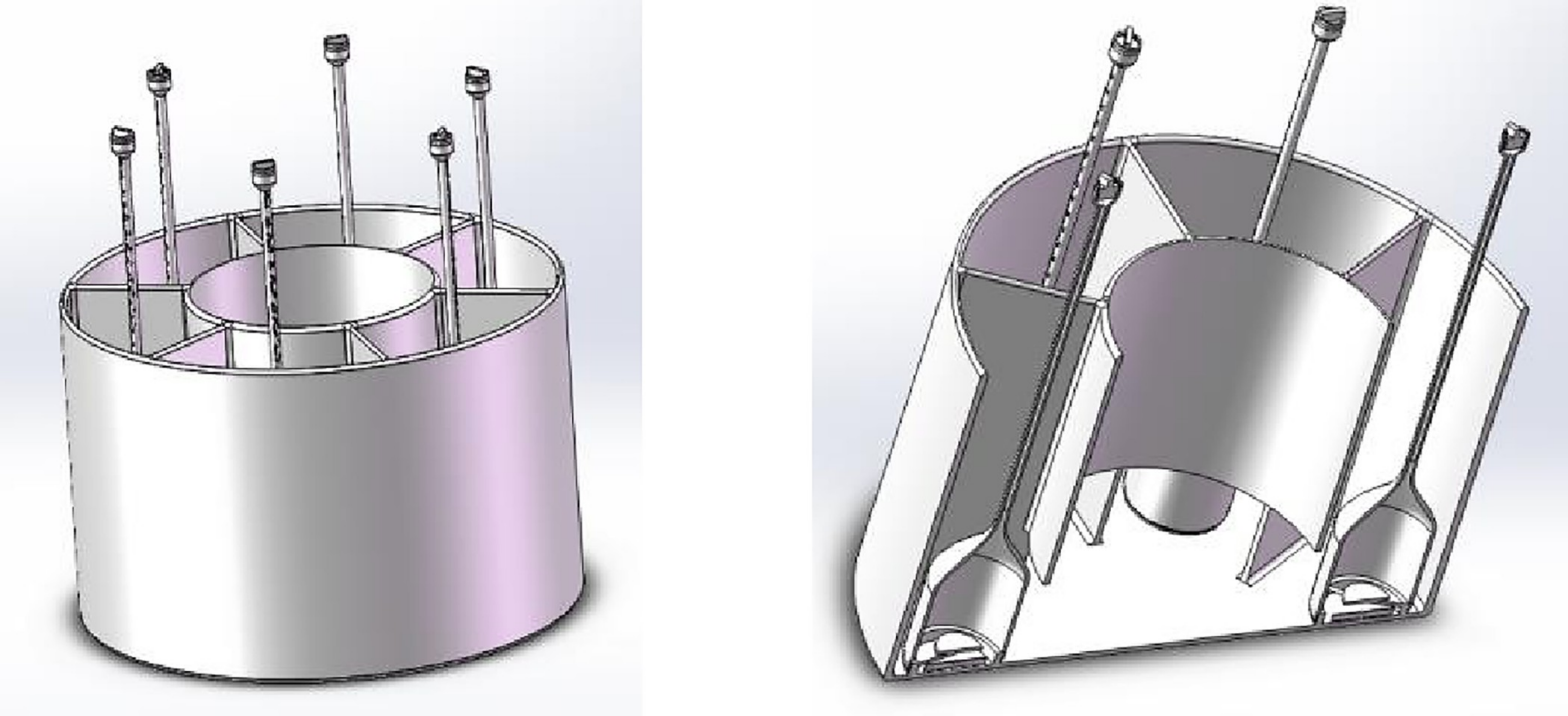
Three-dimensional and sectional drawing of a dynamic/static imbibition experimental device.

**Fig 5:**
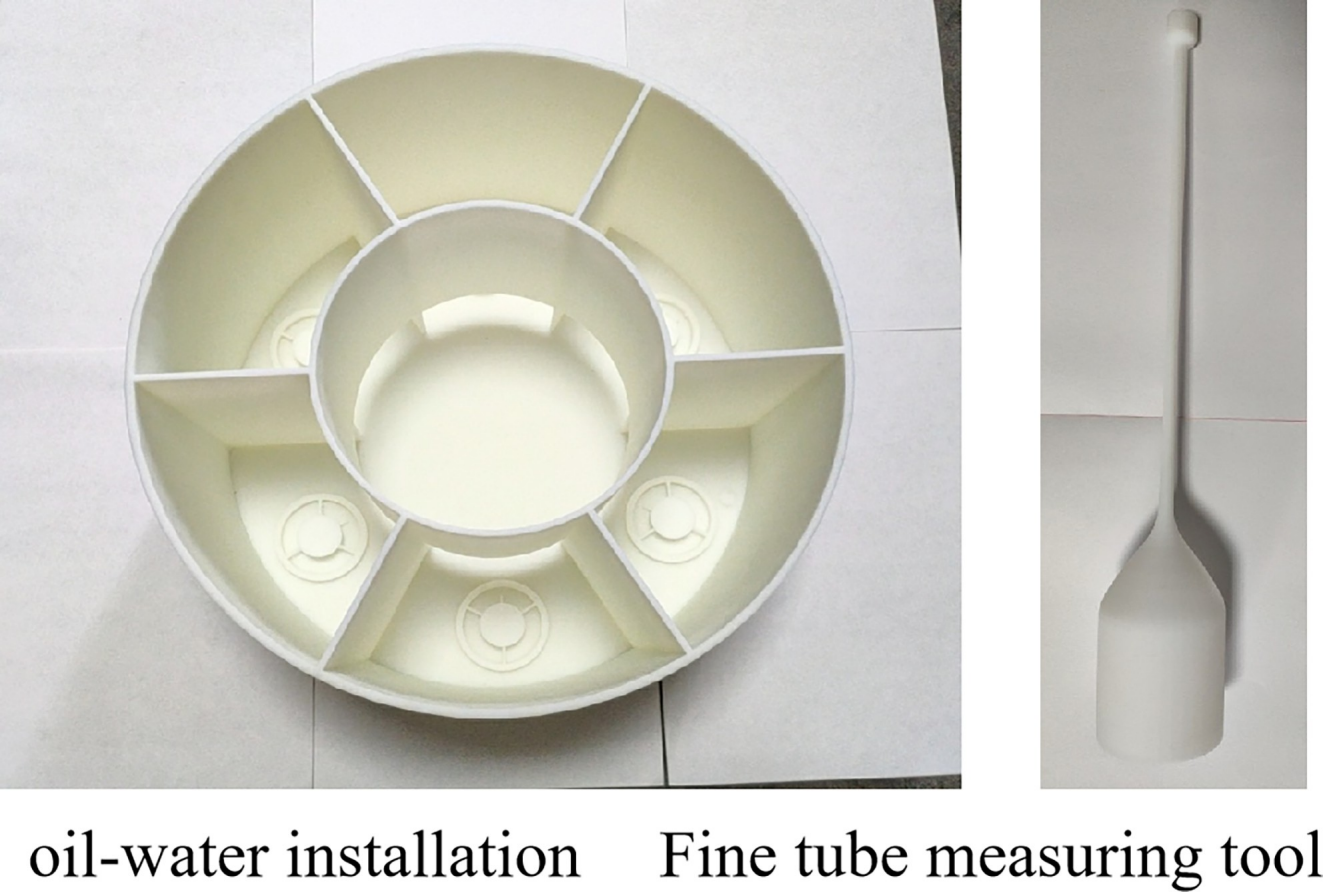
3D printing of physical objects.

The dynamic/static imbibition experimental setup is based on the fact that the replacement fluid is able to flow at a certain rate in fractured media with high permeability and hardly flows at all in bedrock with very low permeability. The main structural components of this dynamic/static imbibition experimental device are six fine tube measuring tools, an oil and water container, six motors, six rock sample support tables, etc., as shown in Figs 1–5.

As shown in Figs 2 and 3, a number of imbibition vessels, independent of each other, are designed in parallel, and a fluid perturbation motor is installed in each imbibition vessel to quantify the fluid flow rate by adjusting the motor rotation speed to simulate the flow of the replacement fluid in the fracture medium. As shown in Figs 1 and 4, each imbibition vessel is designed to contain a fine tube measuring tool with an inverted scale for measuring the volume of oil drained by imbibition. The connecting structure at the bottom of the peripheral container and the center container can realize that the peripheral imbibition container is full of imbibition liquid during the experiment, and the simulated oil and imbibition liquid are stratified on the side of the center container, so as to avoid the possibility that the saturated oil rock samples will be affected by the imbibition liquid by the air entering into the imbibition liquid directly in the process of the experiment.

Through this device, multiple rock samples saturated with oil are subjected to multiple dynamic imbibition experiments at one time according to the dynamic imbibition experimental method adapted by the device, which is convenient for comparing the dynamic imbibition effects under different perturbation conditions in parallel and investigating the influence of the flow of the replacement fluid in the fracture on the imbibition. Turning the motor off with no disturbance of the imbibition fluid is a static imbibition condition. The components of the experimental setup in the following are labeled in Figs 1–5.

### 2.2 Experimental methods for dynamic/static imbibition experiments

1. The test rock sample was vacuumed, saturated with water to calculate the pore volume V_1_, and then saturated with simulated oil to calculate the saturated oil volume V_2_.
2. Add imbibition fluid and simulated oil successively to the center vessel to stabilize the oil-water interface at the upper middle of the inner wall of the center vessel, and make sure that there is no simulated oil in the peripheral imbibition vessel.
3. A rock sample saturated with oil is passed through the center vessel, through the oil-water interface, and placed on the sample support table by the bottom of the cylindrical inner wall.
4. Invert the sample container of the fine-tube measuring tool onto the support table, place a large sealing gasket, and screw it onto the external threaded bracket of the oil and water container to ensure that the sample is in the sample container and not in contact with the wall of the fine-tube measuring tool.
5. Fill the upper inlet of the fine-tube measuring tool with imbibition fluid until the liquid level is at a scale position on the upper scale, and cap the permeate inlet with a threaded cap.
6. Turn on the power motor and adjust it to a set speed to produce some disturbance of the imbibition fluid in the rock sample container. In the static imbibition experiments, the motor speed was set to 0 and the imbibition fluid was not perturbed.
7. Record the volume of simulated oil V_3_, V_4_,… seeped out of the fine tube measuring tool at certain time intervals T until the amount of simulated oil seeped out of the fine tube measuring tool is no longer increasing, and record the final volume of simulated oil seeped out V.
8. The dynamic/static imbibition recovery rate under these conditions is denoted as R = V/V_2_×100%.

### 2.3 Characteristics of the experimental setup

**Fig 6:**
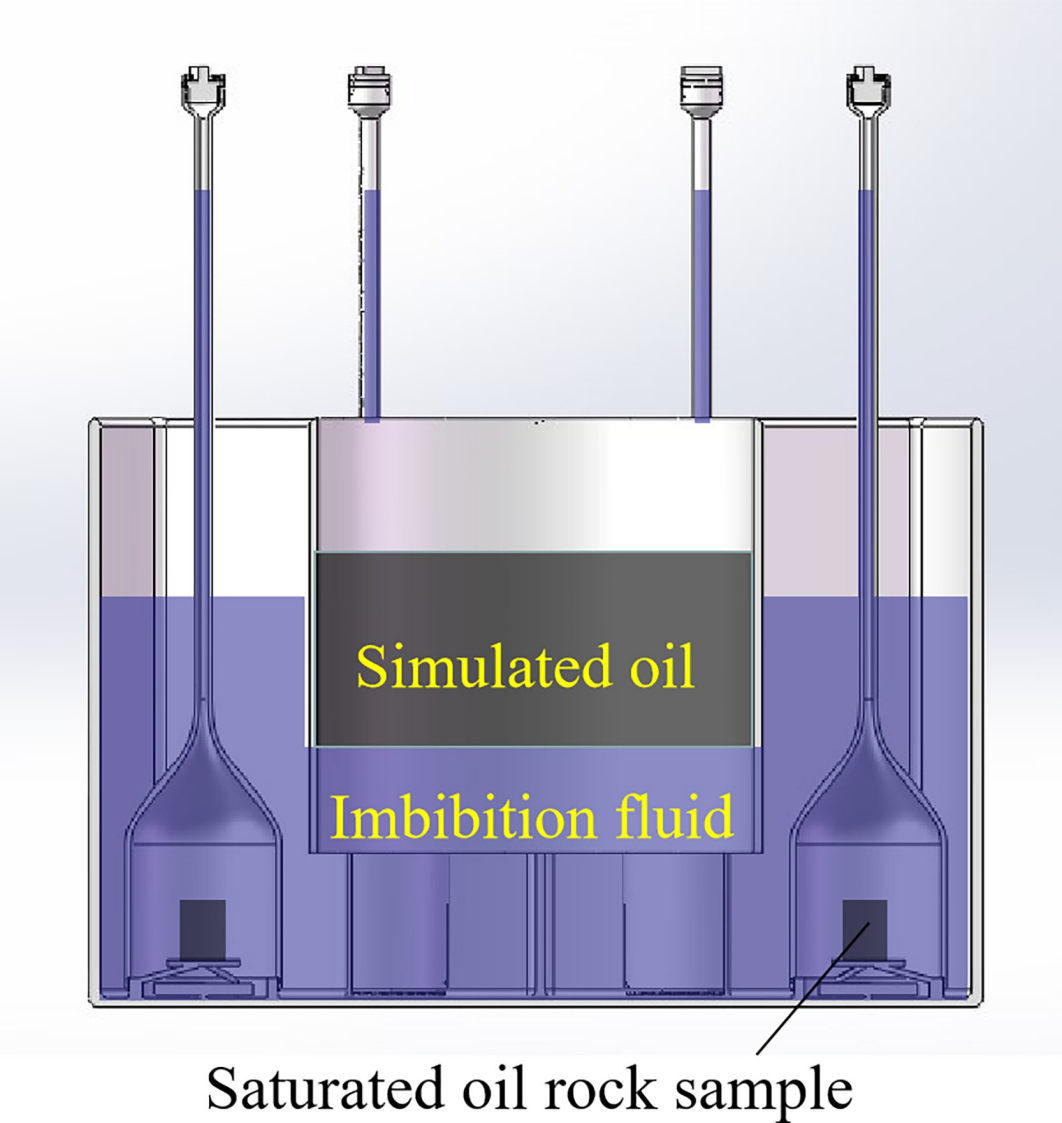
Schematic diagram of device imbibition experimental state.

1. The motor speed controls the flow state of the imbibition fluid. When the motor speed is zero, static imbibition experiments can be conducted. Dynamic imbibition experiments can be carried out with different motor speeds to simulate the effect of different flow rates of the fracture fluid on the imbibition recovery in the fracture matrix system.
2. The peripheral container is connected to the bottom of the central container to realize that the peripheral container side is full of imbibition liquid and the central container side is layered with simulated oil and imbibition liquid, as shown in Fig 6.
3. Before the saturated oil rock samples are placed on the sample support table, they pass through the simulated oil before entering the imbibition fluid, which avoids the possibility of the surface of the rock samples being contaminated by air and affecting the imbibition effect.
4. Parallel design of six sets of fine tube gauges. The static imbibition experiment and the dynamic imbibition experiment with different degrees of disturbance can be carried out at the same time, and it is easy to compare and observe the influence of different degrees of disturbance on imbibition. Multiple sets of imbibition experiments were performed in a single run, increasing the efficiency of the experiments.

### 2.4 Calculation of fracture fluid flow rate and motor speed conversion method

Measurement of fluid flow rates in cracks based on the fracture-matrix model with an example of a specific indoor seepage experiment. The coefficient of variation was 3.4 for the fracture-matrix two-pipe juxtaposition model with a core size of 4.5 cm × 4.5 cm × 30 cm, high and low permeabilities of *K*_*2*_ = 9.5 × 10^−3^μm^2^ and *K*_*1*_ = 2.8 × 10^−3^μm^2^, porosity of 18.2% and 16.4%, respectively, and an injection flow rate of 3.5 ml/min × 2. 17:3 ratio of flow split between high and low permeability cores at steady seepage state.

The high-permeability core was converted to a purely fractured core with only one main fracture, applying the specific parameter data from the example to calculate. Then the fracture width *Δf* for the high permeability core simulation can be calculated from the fracture porosity Formula (1) and the purely fractured rock permeability Formula (2), which is about *Δf* = 371μm; the crack end face overflow area is calculated from Eq (3) as approximately *A* = 16.7 mm^2^; the crack flow rate is calculated from Eq (4) as approximately *Q* = 5.95 ml/min; and the fluid flow velocity in the crack is calculated from Eq (5) to be approximately *v*_*f*_ = 36 cm/min. The diameter of the core vessel of the fine tube measuring tool is 5cm, considering the viscous force of the fluid in the container makes the fluid itself’s disturbing speed much lower than the motor speed (in the case of a lower motor speed and a smaller area of the rotating fan blades, the phenomenon is more obvious). Assuming that the motor speed is about 10 times the fluid’s own disturbing speed, the fluid flow velocity in the crack is converted to the motor speed N by Eq (6), and it is about *N* = 23.2 r/min.


$$\phi_{f}=\frac{\Delta f\times 10^{-3}}{4.5\times 10}$$ (1)

Where *φ*_*f*_ is the crack porosity and *Δf* is the crack width (μm).


$$K_{f}=\frac{\phi_{f}\Delta f}{12}$$ (2)

Where *K*_*f*_ is the crack permeability (cm^2^).


$$A=\Delta f\times 10^{-3}\times 4.5\times 10$$ (3)

Where *A* is the crack end face overflow area (mm^2^) and *Δf* is the crack width (μm).


$$Q=3.5\times 2\times \frac{17}{17+3}$$ (4)

Where *Q* is the crack flow rate (ml/min).


$$v_{f}=\frac{Q}{A}$$ (5)

Where *v*_*f*_ is the fluid flow velocity in the crack (cm/min).


$$N=\frac{v_{f}}{5\times \pi }\times 10$$ (6)

Where *N* is the converted motor speed (r/min).

## 3. Imbibition displacement experiments on extra-low-permeability rocks

The dynamic/static imbibition experimental device was applied to carry out imbibition experiments on extra-low permeability rocks, comparing the static imbibition with the imbibition bottles and the dynamic imbibition with the displacement dynamic imbibition mode, respectively, and examining the effects of air contamination of the saturated oil rock surfaces on the static imbibition and the effects of the replacement factors on the dynamic imbibition. By controlling the motor speed, the fluid flow rate in the crack was simulated to investigate the effect of different crack widths on the imbibition effect.

### 3.1 Selection of imbibition agents

Low interfacial tension conditions facilitate the activation of crude oil, reduce the adhesion work of crude oil on the pore surface, and improve the fluidity of crude oil in the process of rock imbibition production. High interfacial tension and good water-wetting properties are conducive to improving the capillary force and increasing the power of imbibition. Based on the above two considerations, JY-4B, a sulfonate anionic surfactant with good wetting improvement performance, and DHT, a low interfacial tension surfactant, were compounded in a certain ratio. The interfacial chemistry of the solution of the compounded system formulated with highly mineralized simulated water is shown in Table 1. Many reports have suggested that surfactant solution as an imbibition fluid has a superior imbibition effect when the interfacial tension between oil and water is in the order of 10^−2^–10^−1^ mN/m [32–34]. By comparing the values of interfacial tension and contact angle, respectively, compound system 3 was selected as the reagent for the imbibition experiments.

**Table 1:** Interfacial tension and contact angle of the composite system.

Compound System	JY-4B and DHT mass ratio	Surfactant Mass Concentration in imbibition solution	Interfacial tension (10^-2^mN/m)	Contact angle(°)
1	1:2	0.3%	0.45	68.2
2	1:1		2.1	56.7
3	2:1		9.5	52.1

### 3.2 Static imbibition

#### 3.2.1 Experimental materials and instruments

The oil and water characteristics of the Changqing oilfield in China were used as the experimental conditions.Simulated formation water with a mineralization of 28000 mg/L was prepared, and Changqing dehydrated crude oil and kerosene were prepared into simulated oil with a viscosity of 2.4 mPa·s according to a certain ratio. Core column with permeability of 2.3×10^−3^μm^2^ and size of 4.9cm2×5cm, compounding system 3, dynamic/static imbibition experimental setup, imbibition bottle, and stainless steel long elbow tweezers.

#### 3.2.2 Experimental steps

The key to the experimental process lies in the procedure of introducing the rock samples into the experimental setup, and the main experimental steps are as follows:.

1. The core was cut into small core columns about 5 cm high, dried, and vacuumed to saturate simulated water to calculate porosity.
2. A small core column saturated with water is placed in a core gripper and saturated with simulated oil, and oil saturation is calculated.
3. Compound System 3 with a mass concentration of 0.3% was prepared from simulated water as an experimental imbibition solution. It was vacuumed for 2 hours to eliminate the dissolved gas in the aqueous solution and left for use.
4. Refer to "2.2 Experimental Methods for Dynamic/Static Imbibition Experiments" for the subsequent steps of the imbibition experiment using the dynamic/static imbibition experimental setup.

#### 3.2.3 Experimental scheme

Apply the dynamic/static imbibition experimental device and the conventional imbibition bottle to carry out static imbibition experiments, respectively, and make a comparison. Examine the differences in the state of the rock samples and the imbibition recovery rate during the experimental process of the two devices, and the specific scheme design is shown in Table 2.

**Table 2:** Static imbibition displacement experiment scheme.

Scheme	Experimental setup	Imbibition mode	RPM Setting	Imbibition fluid	Core	T
1	Imbibition bottle	Static imbibition	—	Compound system 3 with a concentration of 0.3%	Core columns with permeability of about 2.3 × 10^−3^μm^2^	60°C
2	dynamic/static imbibition experimental device		N = 0			

#### 3.2.4 Experimental results and analysis

**Fig 7:**
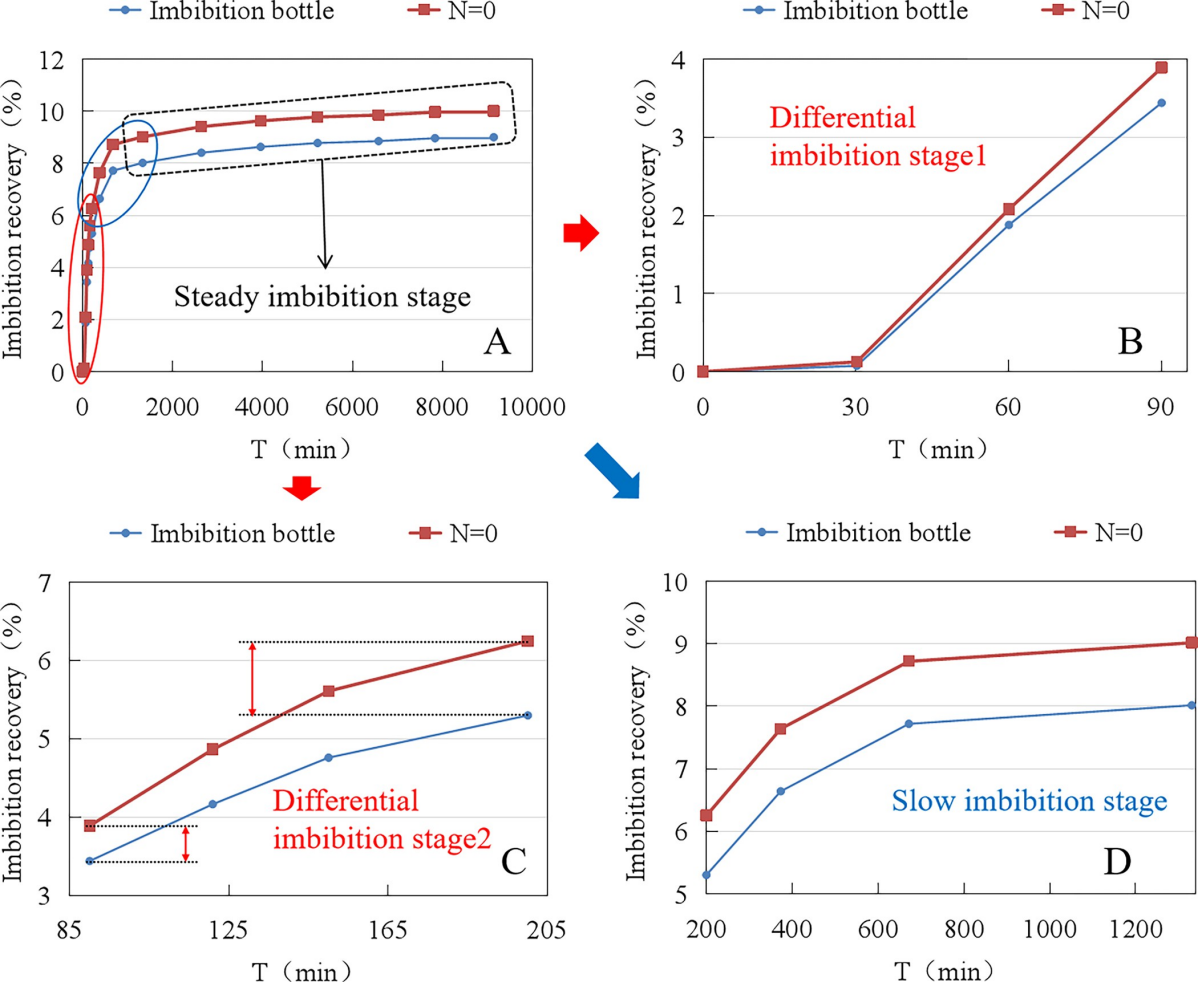
Comparison of static imbibition experimental processes between imbibition bottles and dynamic/static imbibition experimental devices.

As shown in Table 3, the static imbibition recovery rate of the dynamic/static imbibition experimental setup is 1.55% higher than that of the imbibition bottle. As shown in Fig 7, after homogenizing the experimental data, the experimental curves reflect a more pronounced imbibition pattern. The whole imbibition phase was divided into three stages: differential imbibition, slow imbibition and stable imbibition, as shown in Fig 7(A). The imbibition recovery during 0–30 min is close to 0, as shown in Fig 7(B). The growth rate of imbibition recovery was the fastest during 30–200 min, which was close to linear growth, while the difference between the imbibition recovery rates measured by the two devices gradually increased, as shown in Fig 7(B) and 7(C). The growth rate of imbibition recovery rate slowed down significantly during 200–1300 min, and the difference in imbibition recovery rate measured by the two devices was basically constant, as shown in Fig 7(D). After 1300 minutes, the prolonged increase in imbibition recovery measured by both devices was minimal.

**Table 3:** Static imbibition experimental data statistics under different experimental device conditions.

Experimental setup	Core number	Permeability (10^−3^μm^2^)	Porosity (%)	Oil saturation (%)	Imbibition Recovery (%)
Imbibition bottle	45–12	2.21	17.22	61.21	8.51
Dynamic/static imbibition experimental device	46-1-3	2.32	17.18	61.14	10.06

When the core begins to imbibe, oil droplets rapidly appear on the surface and gradually agglomerate to become larger. There is no oil in the upper metering tube until it has increased to the point of detachment from the core surface, and the imbibition recovery rate is counted as zero for an initial period of time. When the oil beads agglomerate and become larger to a certain extent, under the action of buoyancy, they detach from the surface of the core and enter the upper metering pipe, and the imbibition recovery rate begins to grow. Several small air bubbles were found on the surface of the cores in the imbibition bottle during the experiment, while no air bubbles were found on the surface of the cores in the dynamic/static imbibition experimental setup. The presence of air bubbles on the core surface makes the gas/oil, oil/water, and water/gas three-phase interfaces coexist at the same time, and the interfacial tension is complicated, which is an obstacle to water absorption and oil drainage. Comparing the static imbibition experimental process of the two devices, it is believed that the data measured by the dynamic/static imbibition experimental device is closer to the real oil-water imbibition recovery rate.

### 3.3 Dynamic imbibition

Dynamic imbibition experiments are often performed by driving dynamic imbibition: a wire-cut core, either surface "sealed" or unsealed, is placed in a core gripper, and the dynamic imbibition recovery is measured by driving. The process assumes that the injected fluid flows mainly along the cut fracture, that the recovered oil is mainly contributed by dynamic imbibition, and that the final recovery represents dynamic imbibition recovery.

The measured crack width Δf of a core column with a specification of 4.9 cm^2^×5 cm after wire cutting and then buttressing is about 0.1 mm. Assuming that all of the injected fluid flows through the crack in the buttress core, based on Eq (7), the relationship between the flow velocity *v*_*f*_ and the injection flow rate *Q* can be established and calculated.


$$Q=2.5\times \Delta f\times v_{f}$$ (7)

The relationship between *Q* and *N* under the same perturbation conditions of the fluid is established by Eqs (6) and (7), which allow the comparison of the difference in dynamic imbibition recovery between the two dynamic imbibition methods.

#### 3.3.1 Experimental materials and instruments

Simulated formation water with a mineralization of 28,000 mg/L and simulated oil formulated with Changqing dehydrated crude oil and kerosene in a certain ratio, with a viscosity of 2.4 mPa·s. Core column with permeability of 2.3×10^−3^μm^2^ and size of 4.9cm^2^×5cm, compounding system 3, dynamic/static imbibition experimental setup, high temperature and high pressure drive equipment, 5ml small measuring cylinder, PTFE raw tape, and stainless steel long elbow tweezers.

#### 3.3.2 Experimental scheme

Displacement dynamic imbibition mode 1: The arc-shaped sides and ends of the saturated oil wire-cut core were sealed with PTFE raw tape. It was placed in a core gripper, and the imbibition fluid was injected at different flow rates set under 5 MPa confining pressure.

Displacement dynamic imbibition mode 2: The wire-cut cores saturated with oil were not sealed in any way and were placed directly in the core gripper. The imbibition fluid was injected at different flow rates set under 5 MPa confining pressure.

Dynamic/static imbibition experimental device method: the core saturated with oil is placed on the rock sample support table in the imbibition container through the center container, and different motor speeds are set. See "2.2 Experimental Methods for Dynamic/Static Imbibition Experiments" for the specific operation steps.

**Fig 8:**
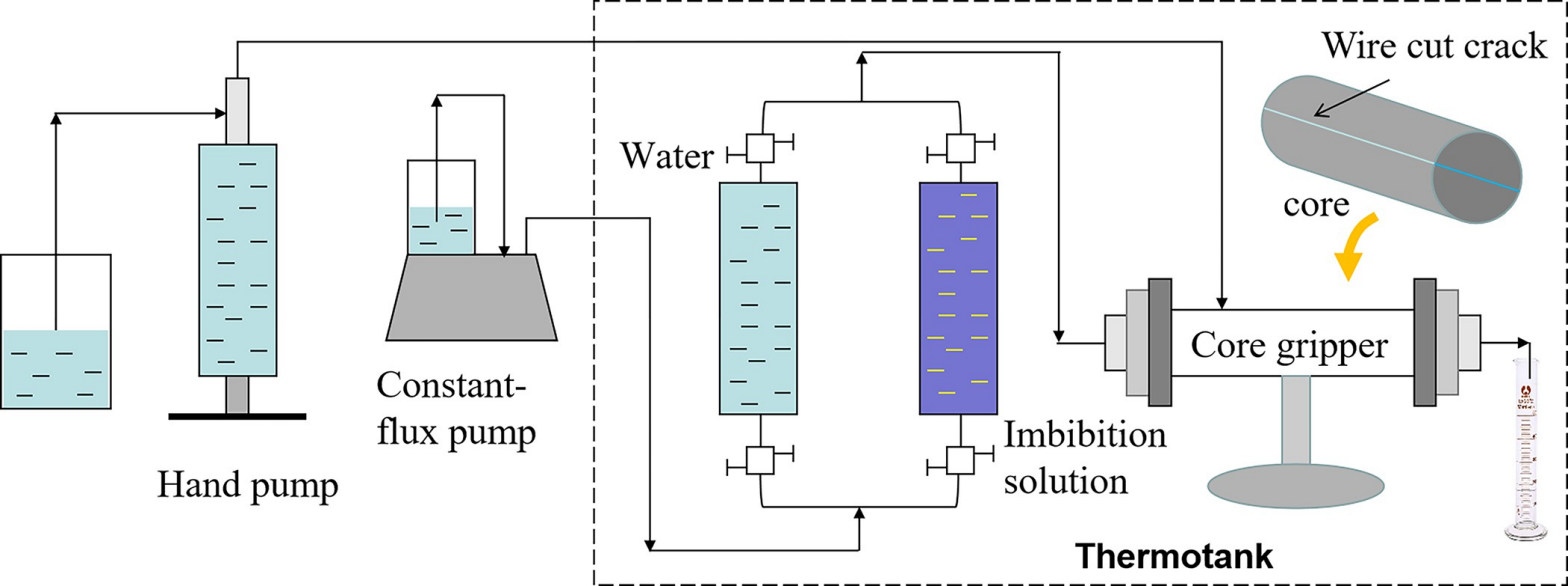
Flow chart of the displacement dynamic imbibition experiment.

To examine the effect of imbibition using different dynamic imbibition experimental methods at fluid flow velocities of 6 cm/min and 18 cm/min, respectively. Combining Eqs (6) and (7), the correspondence between the fluid flow velocity, the motor speed, and the injected flow rate is calculated, as shown in Table 4. The experiments were carried out at 60°C for all three modalities, and the displacement dynamic imbibition experiment is shown in Fig 8.

**Table 4:** Correspondence between fluid flow rate, motor speed, and injection flow.

Flow velocity of the imbibition fluid *v* (cm/min)	6	18
Motor speed of the dynamic/static imbibition experimental device *N* (r/min)	4	11
Injected flow rate of the displacement dynamic imbibition mode 1 *Q* (ml/min)	0.15	0.45
Injected flow rate of the displacement dynamic imbibition mode 2 *Q* (ml/min)		

#### 3.3.3 Experimental results and analysis

**Fig 9:**
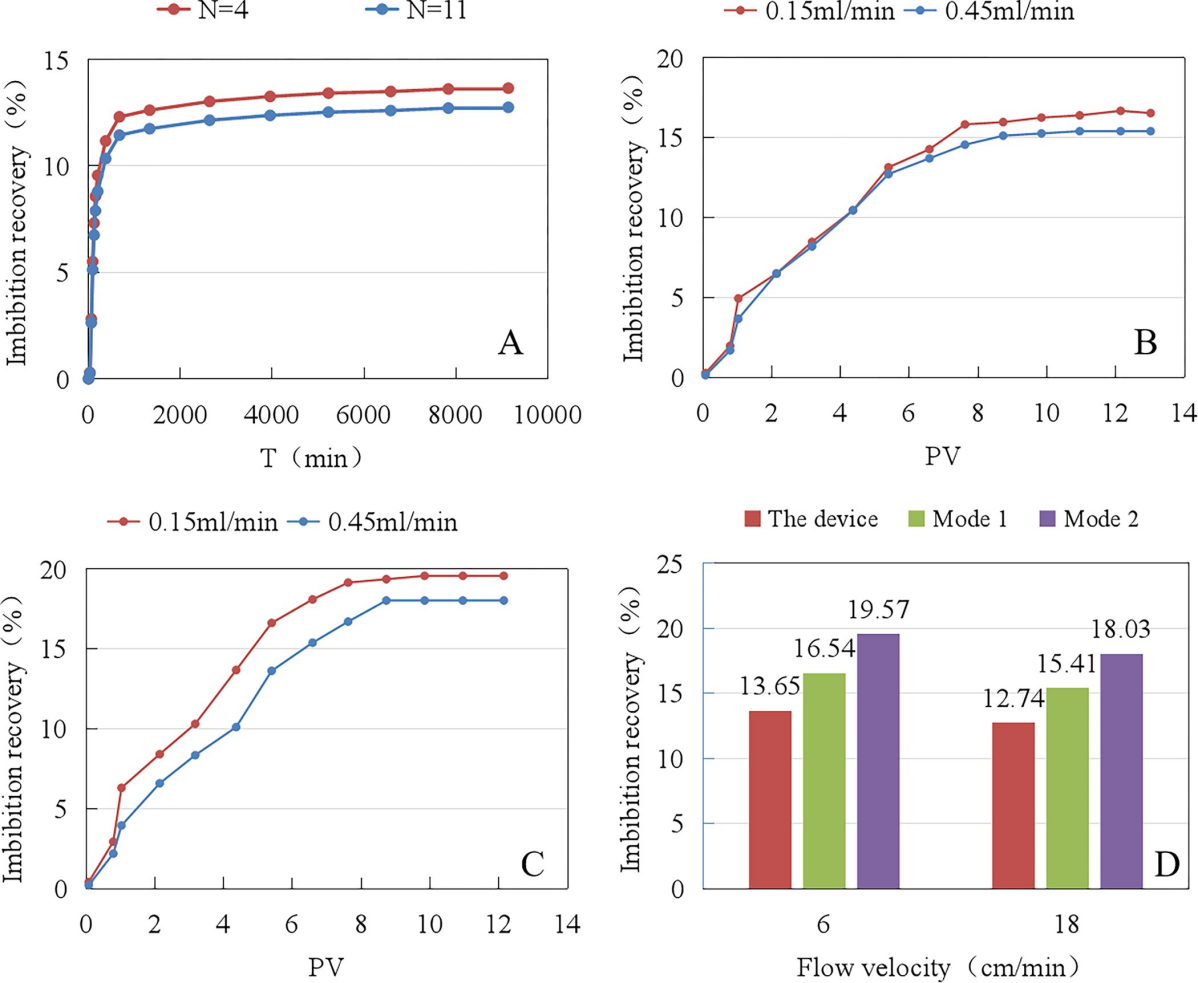
Comparison of imbibition recovery by different dynamic imbibition experimental methods: (A) Dynamic/static imbibition experimental device method; (B) Displacement dynamic imbibition mode 1; (C) Displacement dynamic imbibition mode 2; (D) Comparison of imbibition recovery by different methods.

As shown in Table 5. The dynamic imbibition recovery from the dynamic/static imbibition experimental setup is 3%-6% lower than that of the displacement dynamic imbibition mode. Fig 9(A) shows the dynamic imbibition experimental data from the dynamic/static imbibition experimental setup, which shows that the dynamic imbibition recovery rate gradually increases with the increase in imbibition time, with a rapid increase before 200 min and a slow increase to the extreme value after 200 min. Fig 9(B) and 9(C) shows the experimental data of dynamic imbibition for displacement dynamic imbibition modes 1 and 2, respectively, which show that the dynamic imbibition recovery gradually increases with the increase of the number of injected PVs, with a rapid increase during the period of 0–8 PV and a slow increase to the extreme value after 8 PV. As shown in Fig 9(D), the dynamic imbibition recoveries of the three methods at the same fluid flow velocity are as follows: displacement dynamic imbibition method 2 > displacement dynamic imbibition method 1 > dynamic/static imbibition experimental setup method. The different experimental methods showed higher values for dynamic imbibition recovery with an imbibition fluid flow velocity of 6 cm/min.

**Table 5:** Dynamic imbibition experimental data statistics under different experimental device conditions.

Experiment scheme	Flow velocity	Core number	Permeability (10^−3^μm^2^)	Porosity (%)	Oil saturation (%)	Imbibition Recovery (%)
dynamic/static imbibition experimental device	Low	45–1	2.31	17.12	61.11	13.65
	High	45–2	2.41	17.28	61.24	12.74
displacement dynamic imbibition mode 1	Low	45–3	2.29	17.15	61.15	16.54
	High	45–4	2.38	17.30	61.35	15.41
displacement dynamic imbibition mode 2	Low	45–5	2.27	17.09	61.18	19.51
	High	45–6	2.39	17.32	61.30	18.03

Under the condition of displacement dynamic imbibition modes, due to the presence of external injection pressure, part of the imbibition fluid enters the matrix at that pressure to drive oil, and the amount of recovered oil is contributed by the two dynamics of spontaneous imbibition and displacement. Imbibition from the surface layer of the fracture in the wire-cut core is easy to occur, and the crude oil displaced by imbibition flows out along the fracture at the first time by the driving action. At the same time, a small amount of crude oil in the matrix is driven out by the pressure gradient between the core entrances and exits. The superposition effect of the two is most obvious at the beginning of the experiment, so the imbibition recovery rate rises fastest at the beginning of the experiment. Although the displacement dynamic imbibition method 1 sealed the core sides and ends with PTFE raw tape, the imbibition fluid would enter the matrix to drive from the simulated fractures under the influence of injection pressure, so the overall recovery was slightly higher than that of the dynamic/static imbibition experimental setup method. There is no sealing on the core surface in displacement dynamic imbibition mode 2, and the imbibition fluid will enter the matrix to drive oil from the core end face and simulated fractures under the influence of injection pressure, so the overall recovery is slightly higher than that of displacement dynamic imbibition mode 1. By comparing the imbibition recovery of three dynamic imbibition methods, it is concluded that the dynamic/static imbibition experimental setup method can effectively avoid the effect of injection pressure and reflect the effect of a single flow velocity on imbibition.

### 3.4 Influence of fracture width on dynamic imbibition

During the development of extra-low-permeability fractured reservoirs, it has been shown that the flow velocity of the replacement fluid is highest in the main fracture, followed by intermediate fractures and microfractures [35]. Different fracture widths correspond to different flow velocities, and the fracture fluid flow velocity affects the imbibition in the fracture matrix system. By setting the motor speed to perturb the imbibition fluid, the flow state of the fluid in the cracks with different crack widths is simulated, and the effect of different crack widths on the imbibition effect is investigated.

Extra-low-permeability feature reservoirs with fracture widths mainly in the range of 10–300μm [36,37]. Experimentally set the upper limit of motor speed to 30 r/min. In order to test the effect of different fluid flow velocities in the crack on the effect of imbibition, it is assumed that the crack width is proportional to the fluid flow velocity in the crack, and the corresponding relationship between the crack width and the fluid flow velocity in the crack is converted to the corresponding relationship between the crack width and the motor speed by applying the "2.4 Calculation of fracture fluid flow rate and motor speed conversion method," as shown in Table 6. The motor speed is rounded up to the nearest integer for the purpose of device parameterization.

**Table 6:** Mapping the relationship between crack width and motor speed.

*△f* (μm)	371	300	200	100	50	10
Converted motor speed (r/min)	23.2	18.8	12.5	6.3	3.1	0.6
Motor speed in integers (r/min)	23	19	13	6	3	1

#### 3.4.1 Experimental materials and instruments

Simulated formation water with a mineralization of 28,000 mg/L was prepared, and Changqing dehydrated crude oil and kerosene were formulated into simulated oil with a viscosity of 2.4 mPa·s in a certain ratio. Core column with permeability of 2.3×10^−3^μm^2^ and size of 4.9cm^2^×5cm, compounding system 3, dynamic/static imbibition experimental setup, and stainless steel long elbow tweezers.

#### 3.4.2 Experimental scheme

Combined with the correspondence between crack width and motor speed, the design of the experimental scheme is shown in Table 7. For experimental methods, refer to "2.2 Experimental Methods for Dynamic/Static Imbibition Experiments.".

**Table 7:** Imbibition experiment scheme.

Scheme	RPM Setting	Simulated crack width*△f* (μm)	Experimental setup	Imbibition solution	Core	T
1	N = 1	10	Dynamic/static imbibition experimental setup	Compound system 3 at 0.3% by mass	Core columns with a permeability of about 2.3 × 10^−3^μm^2^	60°C
2	N = 3	50				
3	N = 6	100				
4	N = 13	200				
5	N = 19	300				

#### 3.4.3 Experimental results and analysis

As shown in Table 8, the dynamic imbibition recovery showed a trend of first increasing and then decreasing as the motor speed increased, and the final dynamic imbibition recovery reached more than 13% when N = 3 r/min and N = 6 r/min, which was significantly higher than the other speed conditions.

**Table 8:** Dynamic imbibition experimental data statistics under different motor speeds.

Motor speed (r/min)	Core number	Permeability (10^−3^μm^2^)	Porosity (%)	Oil saturation (%)	Imbibition recovery (%)
N = 1	47–1	2.24	17.23	61.28	10.50
N = 3	47–2	2.33	17.19	61.14	13.01
N = 6	47–3	2.17	17.33	60.88	13.80
N = 13	47–4	2.30	17.12	61.64	12.52
N = 19	47–5	2.23	17.29	60.97	11.83

**Fig 10:**
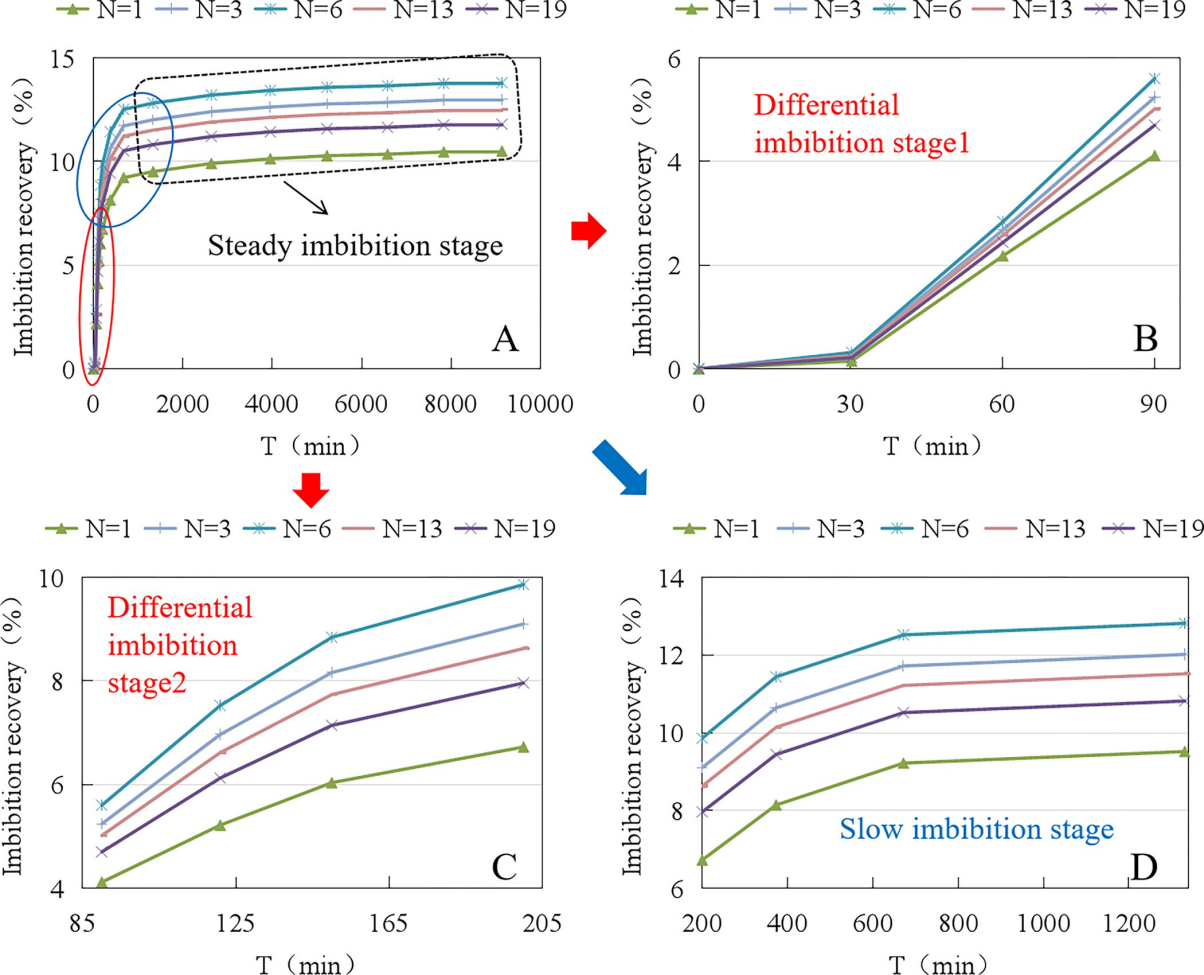
Comparison of dynamic imbibition experimental processes under different motor speeds.

As shown in Fig 10, after homogenizing the experimental data, the dynamic imbibition process similarly reflects three stages: differential imbibition, slow imbibition, and stable imbibition. When N < 6 r/min, the degree of imbibition fluid disturbance increases slightly with the increase in rotational speed. On the one hand, the tendency of diffusion and dispersion of imbibition fluid and surfactant molecules to the core is enhanced, and the surfactant is more likely to produce a concentration gradient in the surface layer of the core, which induces the Marangoni effect, enhances the deformation ability and mobility of the oil droplets in the process of imbibition, and is conducive to the enhancement of the rate of oil-water displacement. On the other hand, the moderately disturbed imbibition fluid can easily make the oil droplets adhering to the core surface agglomerate, become larger, and detach from the core surface, which is conducive to reducing the resistance of oil discharge from the core. When N > 6r/min, the degree of imbibition fluid disturbance is greater, the inertia force is enhanced, and the fluid is more likely to follow the flow law in the space of the large container, which leads to a weakening of the tendency of the imbibition fluid and surfactant molecules to diffuse and disperse into the core, so the oil-water replacement rate is reduced. Although the violent disturbance is more likely to make the oil droplets attached to the core surface agglomerate and become larger, detach from the core surface, and reduce the oil drainage resistance, the weakening of the tendency of the imbibition fluid and surfactant molecules to diffuse and disperse into the core obviously plays a greater role.

The crack widths corresponding to N = 3 r/min and N = 6 r/min are Δf = 50 μm and Δf = 100 μm, respectively, so the imbibition of the fracture-matrix system is more likely to occur near the cracks with a crack width in the range of 50–100μm. Increasing the fracture density in extra-low-permeability reservoirs with fracture widths in the range of 50–100 μm is an important condition for efficient imbibition development in this type of reservoir.

The macroscopic volume of the actual low-permeability oil reservoir is large, and the pore and fracture sizes in it are mostly nano- and micrometer in size and the structure is complex and variable. The dynamic and static imbibition experiments on small-sized cores reflect the limitations of the imbibition characteristics and their influencing factors. It is desirable to combine a large amount of actual reservoir imbibition oil recovery data with statistics and subdivisions of oil and gas reservoir categories to differentiate the macro-controlling factors affecting imbibition oil recovery so as to improve the application effect of imbibition oil recovery in the development of extra-low permeability fractured reservoirs.

## 4. Conclusion

1. A device and its experimental method were designed to be able to perform dynamic and static imbibition experiments simultaneously by adjusting the motor speed. The bottom connection of the device prevents saturated oil rock samples from entering the imbibition fluid directly by air.
2. Applying the data from indoor seepage experiments in conjunction with a pure fracture rock model, a method for measuring the fluid flow rate in a fracture and converting the motor speed is proposed.
3. Under the same experimental conditions, the static imbibition recovery rate measured by the dynamic/static imbibition experimental setup was 1.55 percentage points higher than that of the imbibition bottle, and it mainly occurred in the differential imbibition stage. The dynamic imbibition recovery measured by the device is 3–6 percentage points lower than that of the displacement dynamic imbibition mode, reflecting the effect of a single flow rate condition on imbibition.
4. The results of dynamic imbibition experiments with the dynamic/static imbibition experimental device show that imbibition in low-permeability fractured reservoirs is more likely to occur at the fractures in the interval of 50–100 μm of fracture width.
